# Fibrotic alterations in human annulus fibrosus correlate with progression of intervertebral disc herniation

**DOI:** 10.1186/s13075-021-02690-w

**Published:** 2022-01-17

**Authors:** A. L. Castro, C. Ribeiro-Machado, C. M. Oliveira, G. Q. Teixeira, C. Neidlinger-Wilke, P. Pereira, R. Vaz, M. A. Barbosa, R. M. Gonçalves

**Affiliations:** 1 i3S – Instituto de Investigação e Inovação em Saúde, Universidade do Porto, Porto, Portugal; 2 INEB – Instituto de Engenharia Biomédica, Universidade do Porto, Porto, Portugal; 3 ICBAS – Instituto de Ciências Biomédicas Abel Salazar, Universidade do Porto, Porto, Portugal; 4 ESS - Escola Superior de Saúde, Instituto Politécnico do Porto, Porto, Portugal; 5 Institute of Orthopaedic Research and Biomechanics, Trauma Research Centre, Ulm University, Ulm, Germany; 6 Departamento de Neurociências Clínicas e Saúde Mental, Faculdade de Medicina, Universidade do Porto, Porto, Portugal; 7 Hospital CUF, Porto, Portugal

**Keywords:** Annulus fibrosus, Disc herniation, Fibrosis, Extracellular matrix

## Abstract

**Background:**

Intervertebral disc (IVD) herniation is characterized by annulus fibrosus failure (AF) in containing the nucleus pulposus (NP). IVD herniation involves cellular and extracellular matrix (ECM) alterations that have been associated with tissue fibrosis, although still poorly investigated.

**Methods:**

Here, fibrotic alterations in human AF were evaluated, by characterizing the herniated ECM. Human AF samples (herniated lumbar IVD (*n* = 39, age 24–83) and scoliosis controls (*n* = 6, age 15–21)) were processed for transmission electron microscopy and histological/immunohistochemical analysis of fibrotic markers. Correlations between the fibrotic markers in AF ECM and the degree of NP containment (protused, contained and uncontained) and patients’ age were conducted.

**Results:**

Our results demonstrate that with herniation progression, i.e. loss of NP containment, human AF presents less stained area of sulphated glycosaminoglycans and collagen I, being collagen I fibres thinner and disorganized. On the other hand, fibronectin stained area and percentage of α-smooth muscle actin+ cells increase in human AF, while matrix metalloproteinase-12 (MMP12) production and percentage of macrophages (CD68+ cells) remain constant. These structural and biochemical fibrotic alterations observed in human AF with herniation progression occur independently of the age.

**Conclusions:**

The characterization of human AF here conducted evidence the presence of fibrosis in degenerated IVD, while highlighting the importance of considering the herniation progression stage, despite the patients’ age, for a better understanding of the mechanisms behind AF failure and IVD herniation.

**Supplementary Information:**

The online version contains supplementary material available at 10.1186/s13075-021-02690-w.

## Background

Intervertebral disc (IVD) disorders are a major cause of low back pain (LBP) [1], often associated with ageing and degeneration [2, 3]. During IVD degeneration, the annulus fibrosus (AF) integrity is compromised and its extracellular matrix (ECM) becomes disorganized, impairing the nucleus pulposus (NP) integrity [1]. IVD bulging may occur, without rupture of the AF, resulting in the so-called contained disc herniation, when NP extrusion is prevented by either the AF or the posterior longitudinal ligament (PLL) (hereby designated by protused and contained hernias, respectively, following the Lumbar Disc Nomenclature: version 2.0) [4]. When AF and PLL rupture occurs, the NP may be extruded to the epidural space [5, 6] (hereby designated by uncontained hernias). IVD degeneration is currently best evaluated through magnetic resonance imaging (MRI) that allows the analysis of both disc morphology and hydration [7], which is clinically assessed by one of the five Pfirrmann grades that classify disc degeneration regarding disc height, structure and signal intensity [7, 8]. Nevertheless, the Pfirrmann grade does not relate to pain symptoms and hernia containment.

IVD degeneration is characterized by biochemical and mechanical alterations, with disorganization and degradation of ECM, and chronic inflammation, with increased expression of pro-inflammatory cytokines [9]. Recently, the dysregulation of the IVD ECM during degeneration has been associated to tissue fibrosis [10–12]. Fibrosis is a pathological event occurring in several organs, characterized by a dysregulation of conventional tissue repair, in response to chronic inflammation, resulting in ECM remodelling and excessive accumulation of specific ECM components, which may alter the biomechanical properties of the tissue [13]. Particularly, ECM alterations are characterized by an increase in collagen I (Col I) content and fibronectin (FN) deposition, accompanied by matrix disorganization, leading to scar tissue formation and eventually resulting in organ dysfunction [10, 14]. Alterations in the concentration and morphology of Col I, collagen II (Col II) and FN have been associated with the progression of human IVD degeneration [11, 15]. In human AF (hAF) tissue, a reduction in Col I has been associated with the degeneration level and ageing [1, 11, 16, 17], suggesting a dual effect of both factors in Col I reduction. Col II has also been shown to be reduced in degenerated IVD, compared to healthy IVD [11, 18, 19] and to increase from low to high degeneration grades (evaluated by Thompson’s scale) [15]. The same trend was observed in herniated IVD increasing from protrusion (bulging condition where the AF remains intact) to extrusion conditions [18]. Nevertheless, to the best of our knowledge, fibrotic alterations have not been systematically investigated in human IVD herniation. Myofibroblasts (MFs) and macrophages are the major contributing cells for the progression of fibrosis [13, 20]. MFs express alpha-smooth muscle actin (α-SMA) [21] and are responsible for extensive ECM deposition, namely collagen, within fibrotic tissue, altering tissue architecture and function [21–23]. In the IVD, upon injury, MFs have been suggested to be involved in the AF repair process [24, 25]. The presence of α-SMA+ cells in IVD herniation has also been studied, being increased in degenerated and extruded IVDs, both in human and rat [12]. Macrophages regulate fibrosis since they are high producers of transglutaminases and matrix metalloproteinases (MMPs) [21, 26]. Their presence in herniated IVDs has been extensively reported [27, 28], as well as MMPs, but only MMP12 has been suggested as fibrotic marker for IVD degeneration [12, 29].

Here, we hypothesize that AF fibrosis is associated with hernia progression. Therefore, a systematic analysis of structural and biochemical fibrotic markers in hAF from herniated IVDs was performed and correlated the obtained results with the clinical information on hernia containment.

## Methods

### Study population

**Fig. 1:**
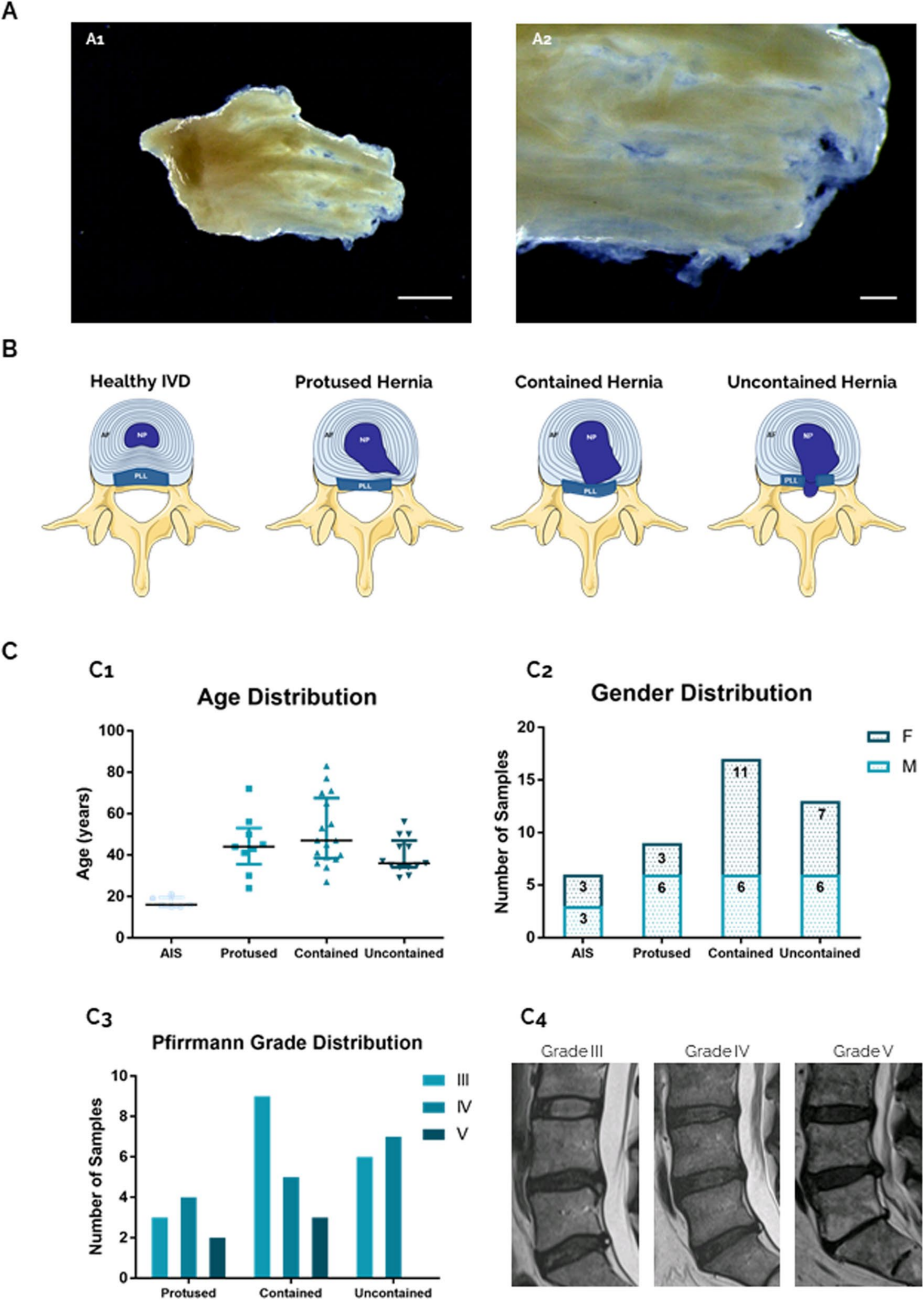
Human AF tissue obtained from LBP patients. **A** Native hAF tissue after macroscopic separation of IVD samples. Scale bar: A1—5 mm; A2—1 mm. **B** Schematic representation of human IVD degeneration/herniation. **C** Clinical data from hAF donors, from scoliosis and LBP patients distributed by hernia containment type (protused, contained, uncontained). C1: age, C2: gender and C3: Pfirrmann grade distribution; C4: representative MRI images of Pfirrmann grade III, IV and V, all in L5-S1 level

Human herniated IVD samples were obtained from patients undergoing microdiscectomy, after informed consent and ethics committee approval, in collaboration with Centro Hospitalar Universitário São João and Hospital CUF (Porto, Portugal). A total of 39 samples (median age 44 (IQR: 35–53 years old)) were dissected to isolate AF tissue (Fig. 1A). Samples were characterized considering the medical information provided regarding hernia type: protused, contained uncontained (Fig. 1B). Control discs without visible morphological alterations were collected from adolescent idiopathic scoliosis (AIS) patients undergoing surgery [*n* = 6, median age 16 (15–19.5 years old)]. Age, gender and Pfirrmann distribution of the donors/samples collected are shown in Fig. 1C. The individual information for all the samples received is presented in Supplemental Table S1.

### Sample collection, processing and analysis

Human IVD samples were collected and incubated with IVD Medium [DMEM (Invitrogen) supplemented with 5% FBS (Biowest), 0.5% fungizone (Invitrogen), 1% penicillin/streptomycin (Invitrogen) and 1.5% of 5 M NaCl/0.4 M KCl solution, for osmolarity adjustment to physiological conditions, as previously described [30, 31] and kept at 4 °C overnight after the surgery procedure until further analysis on the following day (total maximum of 18 h). Samples were macroscopically separated into NP and AF tissue. Fixed hAF tissue was processed for transmission electron microscopy (TEM) analysis, using 3 representative samples from each hernia type condition. Dissected AF samples were fixed (10% neutral buffered formalin), embedded in paraffin and sectioned (3 μm). Sections were stained for Alcian Blue (AB) and Picro-Sirius Red (PSR) to analyse sulphated glycosaminoglycans (sGAG) (blue)/collagens (red) stained area, respectively. Col I, FN, α-SMA, MMP12, Col II and CD68 expression in AF sections was assessed by immunohistochemistry (IHC), using NovolinkTM Polymer Detection Kit (Leica Biosystems), according to manufacturer’s instructions. Both optimized antigen retrieval and antibody dilutions are described in Table 1. The protocols used for the different techniques are extensively described in Supplementary Methods.

**Table 1:** Antibodies select for IHC, antigen retrieval procedure, optimized dilution and incubation period

Antibody	Antigen Retrieval	Incubation	Reference / Brand
Collagen type I	10 mM citrate buffer, pH=6.0, 1’ MW 1,5 U/mL hyalurinodase (Sigma-Aldrich), 45’ 37ºC	Dilution: 1:100 Incubation overnight	600-401-103-0.1 / Rockland Immunochemicals, Inc. Limerick, PA
Fibronectin	10 mM citrate buffer (pH=6.0), 1’ MW 1,5 U/mL hyalurinodase (Sigma-Aldrich), 45’ 37ºC	Dilution: 1:50 Incubation overnight	SC-8422 / Santa Cruz Biotechnology
Alpha Smooth Muscle Actin	10 mM citrate buffer (pH=6.0), 1’ MW 1,5 U/mL hyalurinodase (Sigma-Aldrich), 45’ 37ºC	Dilution: 1:400 Incubation overnight	SC-53015 / Santa Cruz Biotechnology
Matrix Metalloproteinase 12	10 mM citrate buffer (pH=6.0), 1’ MW 1,5 U/mL hyalurinodase (Sigma-Aldrich), 45’ 37ºC	Dilution: 1:400 Incubation overnight	SC-390863 / Santa Cruz Biotechnology
Collagen type II	10 mM citrate buffer (pH=6.0), 1’ MW 20 μg/mL proteinase K (Sigma-Aldrich), 15’ 37ºC	Dilution: 1:100 Incubation overnight	SC-52658 / Santa Cruz Biotechnology
CD68	Tris-EDTA buffer (pH=9.0), 1’ MW	Dilution: 1:100 Incubation 30’	PG-M1 / Dako

*MW* Microwave

### Bioimage analysis

Sections were imaged using a Motic 3.0 camera coupled to a Zeiss Axioskop2 microscope (Carl Zeiss International), except for PSR polarization and CD68 images, which were imaged using an inverted fluorescence microscope (Zeiss Axiovert 200 M).

Bioimage analysis was performed using FIJI free software (v2.0.0). For the AB/PSR staining, area quantification was carried on using 5 images (× 10 magnification) per sample, and a colour threshold was applied to separate areas stained in blue/red. For the polarized light analysis, a mosaic was performed and red/yellow/green collagen fibres were quantified as described above. Regarding IHC quantification, for matrix antibodies (Col I, FN and Col II), 5 images per sample (× 10 magnification) were used for the quantification of the stained area, through threshold application. For cell-specific antibodies (α-SMA, MMP12), 7 images per sample (× 20 magnification) were used, and positive cells were counted using the Cell Counter plugin of FIJI. To better enlighten this process, a schematic workflow can be found in Fig. S1. Finally, for CD68+ area quantification, a mosaic was performed (× 20 magnification), and colour threshold was applied.

### Statistical analysis

Statistical analysis was performed using GraphPad Prism software (v5). Results are presented using median percentage and interquartile ranges. Data normality was assessed using D’Agostino-Pearson normality test. Non-parametric Mann-Whitney tests were performed for comparison between different hernia containment levels. Uni- and multivariate regression analysis was conducted to identify any association between fibrotic markers expression and type of hernia containment. The effect of interaction between age and IVD herniation level on fibrotic markers expression was evaluated in the statistical regression model using statistical software R version 4.0.2 (Project for Statistical Computing). Statistical significance was considered at *p < 0.05, **p < 0.01, ***p < 0.001 and ****p < 0.0001.

## Results

### hAF matrix structural analysis with herniation progression

The hAF was obtained from herniated IVD biopsies collected from patients undergoing microdiscectomy. A macroscopic dissection of IVD samples to isolate the AF tissue was first performed (Fig. 1A). hAF from herniated samples was separated into three categories, according to the information provided by the neurosurgeon: protused, contained and uncontained (Fig. 1B). As controls, hAF was obtained from non-herniated IVD of AIS patients [*n* = 6, median age 16 (15–19.5 years old), 3 males/3 females]. Age, gender and Pfirrmann grade distribution per hernia type can be observed in Fig. 1 C_1_, 1C_2_ and 1C_3_, respectively. Gender distribution is similar in the scoliosis and uncontained groups. In contained hernias, almost two thirds of the samples were from females, and in protused samples, the reverse was observed. Pfirrmann grade distribution among herniated samples shows a low presence of samples with grade V in all categories. In addition, the samples with Pfirrmann grade III and IV are evenly distributed, except in contained hernias, in which samples with Pfirrmann grade III are more numerous (Fig. 1 C_3_).

**Fig. 2:**
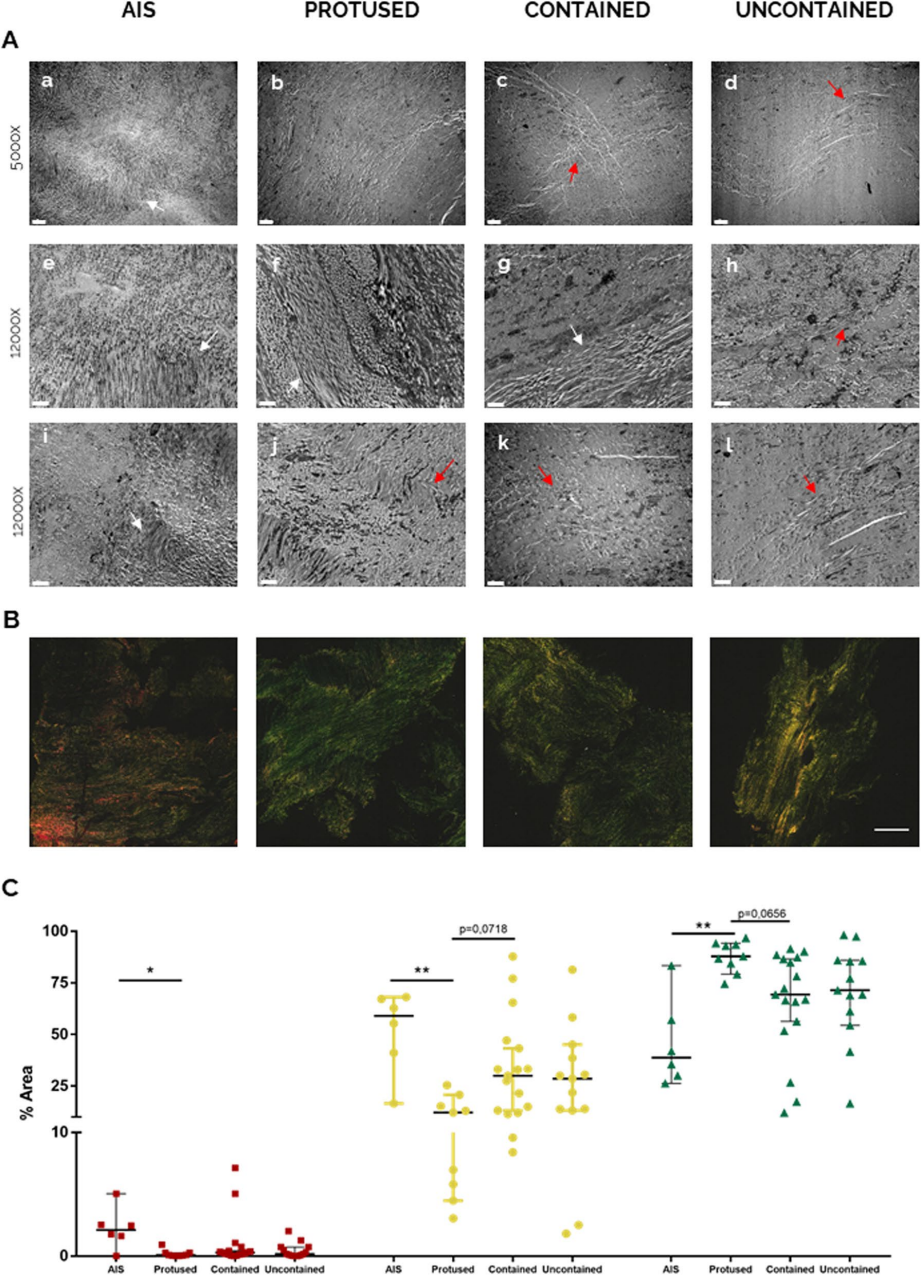
Human AF matrix ultra-structural characterization with herniation progression (*n* = 3). **A** a–d: × 5000 magnification, scale bar: 2 μm; e–l: × 12,000 magnification, scale bar: 1 μm. White arrows point to well organized collagen fibres, while red arrows point to disorganized fibres. **B** Picro-Sirius Red staining under polarized light, scale bar: 500 μm. **C** Quantification of collagen fibres per birefringence colour. Kruskal-Wallis test followed by corrected Dunn’s were performed. *p < 0.05; **p < 0.01

ECM ultra-structural alterations in hAF were first analysed, regarding matrix density, collagen fibres maturation and distribution/orientation. For that, TEM analysis was performed in representative samples from hernias with distinct containment levels (Fig. 2A). Highly organized collagen fibre bundles (white arrows) together with a few disorganized fibres were observed (red arrows), both in scoliosis (Fig. 2A, a, e, i) and in protused hernias (Fig. 2A, b, f, j). In the contained hernias, a disorganized fibre arrangement was more prominent (red arrows), although some organization (white arrows) was still present (Fig. 2A, c, g, k). On the other hand, uncontained hernias exhibit loss of lamellar organization of the fibres, being these randomly dispersed within the tissue (red arrows) (Fig. 2A, d, h, l).

In addition, the analysis of the thickness of collagen fibres was performed by the analysis of PSR staining under polarized light, as previously described [32]. This evaluation allows the identification of collagen fibres thickness, depending on their birefringence under polarized light. The visualization of red and yellow collagen fibres correspond to thicker fibres, while green correspond to thinner fibres, related with more youthful collagens [33]. This analysis is usually associated with Col I and collagen III (Col III) fibres, although, in this study, the fibres are most probably associated with Col I, since Col III is not abundant in AF and is described only as a pericellular protein within the tissue [34, 35]. Nevertheless, this analysis should be performed using second harmonic microscopy [36]. Figure 2B shows representative images of hAF samples stained with PSR and imaged under polarized light, while Fig. 2C shows the proportion of the collagen fibres (in red, yellow and green) for the different conditions analysed. Results clearly show a lower frequency (< 7%) of the red birefringence (thicker fibres) for all the samples, in comparison with the more mature fibres (yellow/green birefringence). Moreover, fibres with red birefringence were reduced in the herniated samples, compared with AIS. On the other hand, the thinner fibres (green) were present in higher proportion (38–86%) in all the conditions analysed, while the frequency of collagen fibres with intermediate thickness (yellow) range from 12 to 60%. Together with thicker fibres, the intermediate size collagen fibres are also reduced in all herniated samples compared with AIS control, more specifically in protused hernias (12%, **p < 0.01). These fibres slightly increase in contained hernias (30%, *p* = 0.0718) compared with protused hernias.

In what concerns thinner collagen fibres, an opposite trend is observed. In this case, the AF from all herniated discs present a higher proportion of green fibres, compared with AIS discs, being this significantly higher in the case of protused hernias (87.85%, **p < 0.01). With herniation progression, the presence of these fibres is slightly reduced (69.35%, *p* = 0.0656 contained hernias). These results suggest an increased synthesis of new collagen fibres in the AF from herniated discs, compared with AIS controls, particularly in protused hernias.

### hAF matrix biochemical analysis with herniation progression

**Fig. 3:**
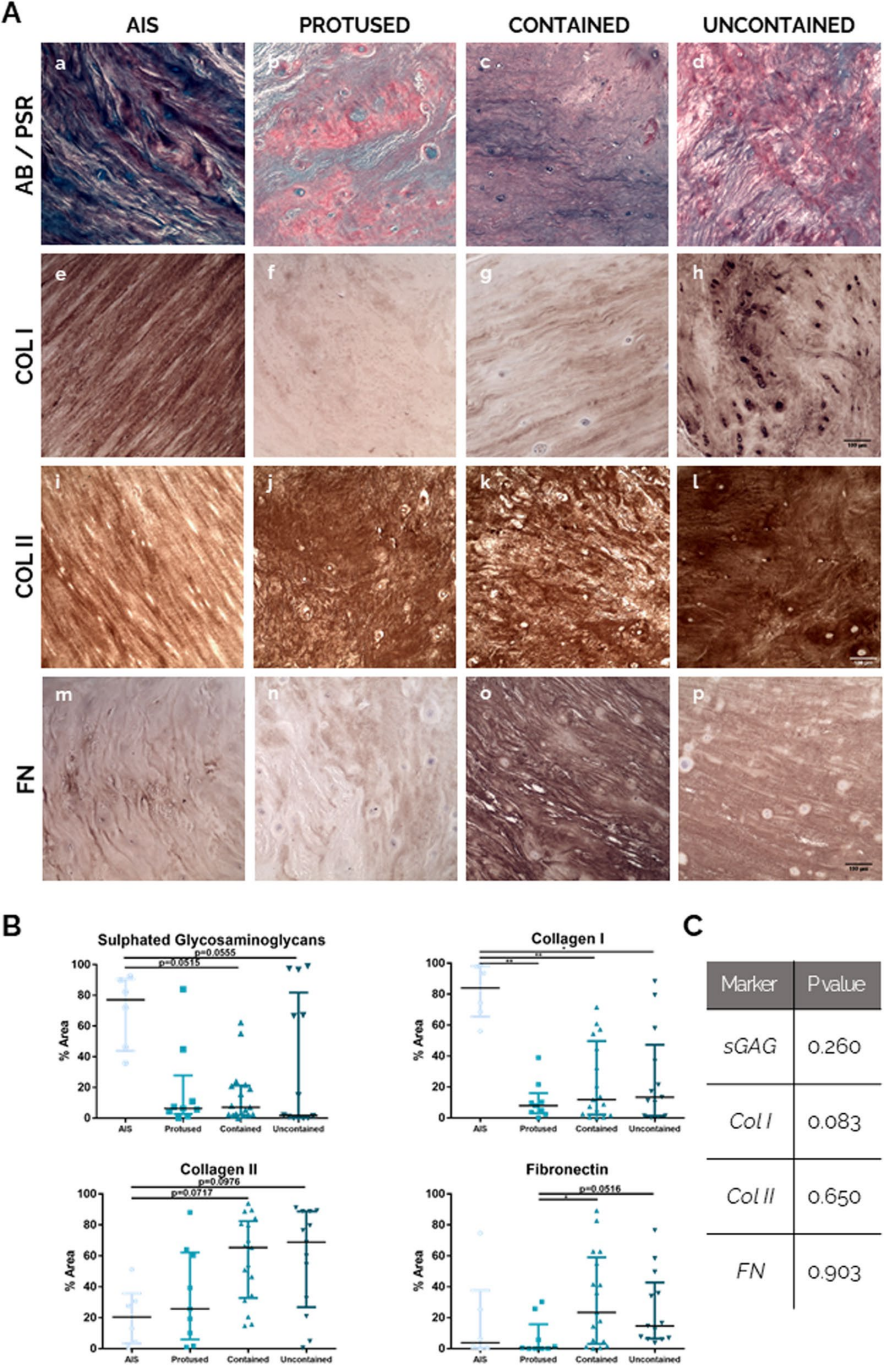
Human AF matrix biochemical characterization with herniation progression. **A** Histological/IHC staining for a–d: Alcian Blue/Picro-Sirius Red, scale bar: 100 μm; e–h: Collagen I, scale bar: 100 μm; i–l: Collagen II, scale bar: 100 μm; m–p: Fibronectin, scale bar: 100 μm. **B** Quantification of each staining per herniation type. Data presented using dot plots, with median and interquartile range. Kruskal-Wallis test followed by corrected Dunn’s were performed. *p < 0.05; **p < 0.01. **C** Multivariate analysis of interaction of hernia containment level with age for each staining

A biochemical analysis of the ECM of hAF was performed by histological/IHC analysis of Col I, Col II, FN and sGAG, generally lost during fibrosis (Fig. 3A) (low magnification images are presented in Supplementary Figure S2). From the images, the percentage of staining area was quantified and the results are presented as the median percentage of area or positive cells and respective interquartile range for each marker (Fig. 3B). Figure 3A (a–d) shows representative images for sGAG staining (blue) in the hAF of different samples. By AB/PSR staining, it can be observed a clear higher stained area of sGAG (blue) in hAF from most AIS samples [77.1% (43.8–90.6)] compared to all herniated samples, particularly in contained hernias [7.1% (2.1–21.3), *p* = 0.0515] and uncontained hernias [1.78% (0.2–81.9), *p* = 0.0555].

Regarding the expression of Col I (Fig. 3A (e–h) and 3B), it is highly present in AF tissue from AIS samples (Fig. 3A, e) [84.0% (65.4–98.1)] and significantly decreased in the AF of all herniated samples [8.0% (3.1–16.0), **p < 0.01, in protused hernias; 11.9% (2.2–49.7), **p < 0.01, in contained hernias; and 13.5% (1.4–47.4) *p < 0.05, in uncontained hernias] (Fig. 3A, f–h). Col II stained area was also assessed (Fig. 3A (i–l) and 3B), showing an opposite trend of Col I. The stained area of Col II is lower in hAF from AIS samples [20.5% (3.5–35.8)] (Fig. 3A, i) increasing in hAF from herniated samples (Fig. 3A, j–l). This increase is close to significant in hAF of contained hernias [65.4% (32.8–82.5), *p* = 0.0717] and uncontained hernias [68.9% (26.8–88.8), *p* = 0.0976].

Relatively to FN expression (Fig. 3A (m–p) and 3B), the results show low and heterogeneous FN expression in protused hernias [0.5% (0.04–15.8)], with 2 (out of 9) samples showing higher FN stained area (about 25.8 and 30.3%). Nevertheless, FN expression tends to increase in hAF from herniated IVD, being significantly higher in contained hernias samples [23.5% (3.2–59.0), *p < 0.05], and in uncontained samples [14.8% (6.6–42.8), *p* = 0.0516].

In addition, to discard that the differences observed might be due to age differences, the interaction between the variables “age” and “hernia containment” for all the markers analysed was addressed using a multivariate analysis. The *p* values obtained (Fig. 3C) demonstrate an absence of interaction between the variables “age” and “herniation progression” (*p* > 0.05) for sGAG, Col I, Col II and FN presence in hAF, reinforcing that the differences in the biochemical composition of hAF ECM with herniation progression are not due to age differences between the different donors.

**Fig. 4:**
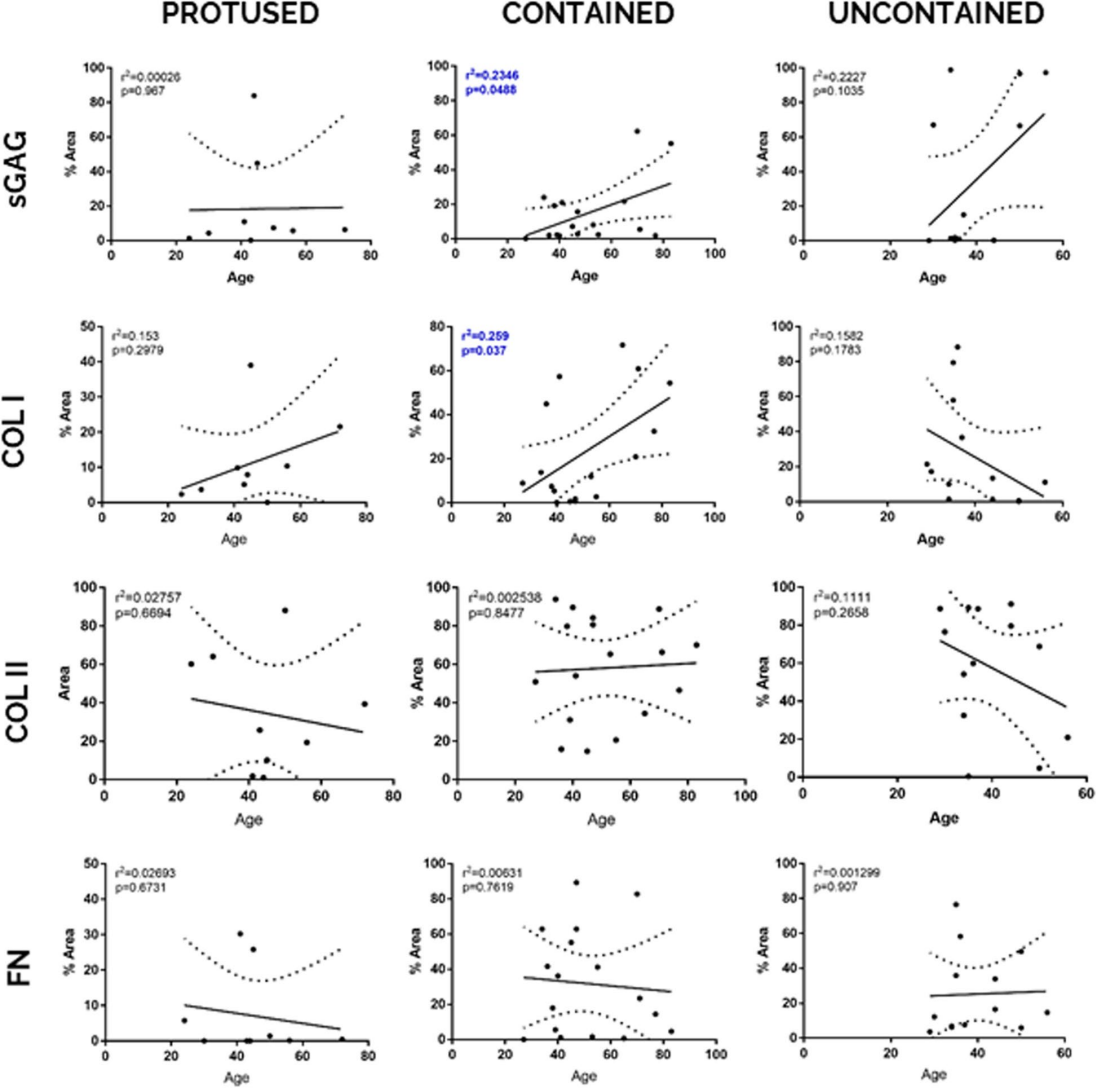
Correlation of expression levels of hAF matrix components with age, within each herniation type. Data presented using dot plots, with 95% confidence intervals and indication of *r*^2^ and *p* value for each linear regression

Moreover, for each herniation stage (protused, contained and uncontained), a linear regression between hAF ECM stained area and donor age was performed (Fig. 4). With this analysis, it is possible to verify a positive correlation for sGAG and Col I with ageing only in the group of hernias contained by PLL (*r*^2^ = 0.2346, *p* = 0.0488 for sGAGs and *r*^2^ = 0.259, *p* = 0.037 for Col I, respectively), suggesting that, within this herniation containment level, Col I and sGAG increase with ageing.

### hAF fibrotic analysis with herniation progression at the cell level

Additionally, relevant cellular markers to tissue/IVD fibrosis (α-SMA+ cells, MMP12+ cells and CD68) were also evaluated in hAF samples by IHC analysis.

**Fig. 5:**
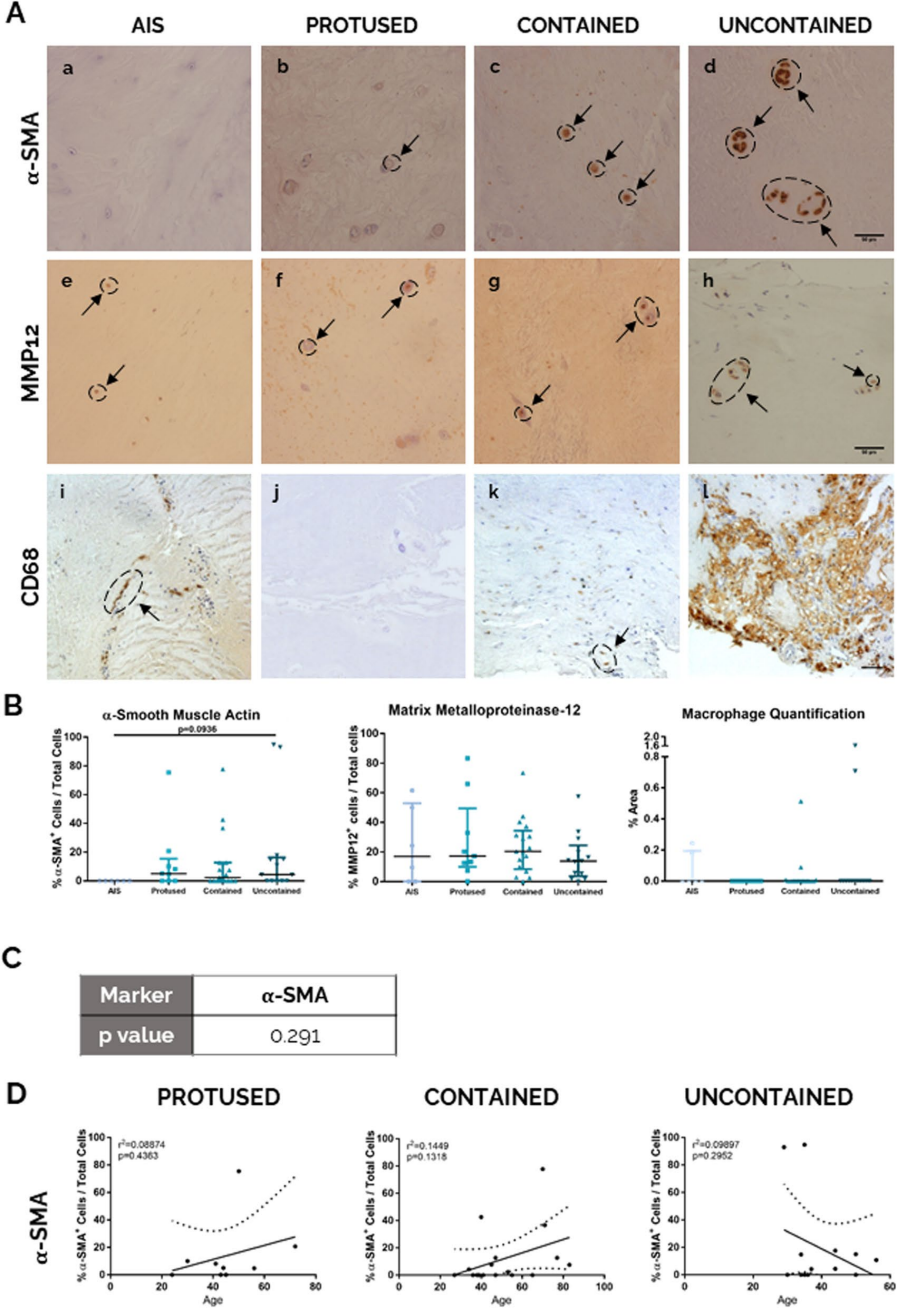
Human AF fibrotic analysis at the cell level with herniation progression. **A** IHC staining for a–d: α-SMA, scale bar: 50 μm; e–h: MMP12, scale bar: 50 μm; i–l: macrophages (CD68) scale bar: 50 μm. **B** Quantification of each staining per herniation type. Data presented using dot plots, with median and interquartile range. Kruskal-Wallis test followed by corrected Dunn’s were performed. *p < 0.05; **p < 0.01. **C** Multivariate analysis of interaction of hernia containment level with age for α-SMA. **D** Correlation of α-SMA staining quantification with age for each herniation type. Data presented using dot plots with 95% confidence intervals and indication of *r*^2^ and *p* value for each linear regression

The α-SMA^+^ cells were absent from hAF of AIS samples (Fig. 5A (a–d) and 5B), as well as in a high percentage of herniated samples (in 33.3% of the protused hernias, 47% of the contained hernias and 38.5% of the uncontained hernias), suggesting a high heterogeneity of the α-SMA expression in hAF. Nevertheless, in the samples presenting α-SMA+ cells, an increased expression of α-SMA in the AF from herniated tissues was observed, ranging from 2.27 to 94.71%, when compared to AIS samples.

In what concerns MMP12 (Fig. 5A (e–h) and 5B), another marker associated with IVD fibrosis, heterogeneity was also observed, since MMP12+ cells were absent in 33.3% of AIS samples, in 11.1% protused hernias, in 5.9% of contained hernias and in 9.1% of uncontained samples. No differences were observed regarding the presence of MMP12 in the different herniated conditions, with most samples exhibiting MMP12+ cells below 50%.

Furthermore, macrophage presence in hAF samples was also assessed by the expression of CD68^+^ cells (a common marker used for macrophages) (Fig. 5A, i–l, and Fig. 5B). CD68+ cells were observed in 33% of AIS samples, in 24% of contained hernias and in 11.7% of uncontained samples, but not in protused hernias (Fig. 5A,i–l). Macrophages were present in AF tissue both, dispersed (Fig. 5A, k) and agglomerated (Fig. 5A, l), the last predominantly in hAF tissue borders (Fig. 5A, l). Due to this observation, macrophage infiltration area was quantified (Fig. 5B), instead of percentage of positive cells. Nevertheless, the quantification of CD68+ area revealed no significant differences between hernia conditions.

As previously described for ECM markers, the interaction between the variables “hernia containment” and “age” was addressed for α-SMA expression using a multivariate analysis (Fig. 5C), but no interaction was found between the two variables (*p* value = 0.291). Moreover, no linear correlations were found between the increase of α-SMA expression with ageing for each hernia containment group.

## Discussion

The AF plays a functional and crucial role in IVD homeostasis since it is responsible for the containment of the NP, preventing its extrusion. A pro-inflammatory environment and extensive mechanical stresses can weaken AF mechanical properties [37], ultimately leading to IVD herniation. But the mechanisms behind IVD herniation and AF disruption remain unclear. The IVD, and particularly the AF ECM, undergoes structural, biochemical and biomechanical alterations that have been studied in different models, as canine, rabbit, mice or bovine [38–41]. IVD degeneration has been associated with tissue fibrosis [10–12], but this has been poorly addressed. It is crucial to understand tissue fibrosis in the IVD, so we could disclose novel cellular and molecular cell targets to stop/revert IVD herniation. Taking this into account, this study aimed to evaluate the fibrotic alterations of AF with hernia progression (here referred to the medical evaluation of hernia containment levels).

Currently used in IVD clinical evaluation, Pfirrmann’s MRI-based classification lacks suitability to assess herniation progression, among other problems [7, 8]. Although considering the structure, brightness and height of the disc [8], Pfirrmann grading system does not distinguish disc herniation progression. For example, in this study, only 5 samples were described as Pfirrmann grade V, characterized by the collapse of IVD to the epidural space [8], but 13 samples were identified as extruded to the epidural space by the surgery team. It is important to combine different classification systems that embrace both image analysis and clinical evaluation, to a more accurate definition of each herniation condition. In the future, systemic markers would be important to improve the accurate diagnosis of IVD herniation progression stages.

The ECM of hAF was first analysed regarding its ultra-structure and biochemical composition. Matrix disorganization and impairment in the AF tissue are characteristics of IVD degeneration and herniation. As expected, in the most severe herniation stage, a more disorganized matrix was found. A low percentage of thicker (more mature) collagen fibres (red) was revealed in all samples. A higher amount of thinner collagen fibres (green birefringence) present within the herniated tissue was observed, suggesting recent ECM remodelling.

Regarding ECM biochemical alterations of hAF, this work used a panel of fibrotic ECM-related markers (Col I, Col II, FN, sGAG) and compared AF from herniated IVD with AF from AIS patients, as control group, due to the impossibility of obtaining human adult healthy IVD. A tendency for lower sGAG stained area in AF tissue from contained hernias and uncontained compared to AIS samples was observed. These changes are in line with the loss of sGAGs previously reported for IVD degeneration in human, as well as in canine and rabbit models [16, 41–43]. Regarding Col I, a decreased expression in herniated IVD was observed compared with AIS samples but increasing with herniation progression. The reduction of Col I stained area in AF from herniated tissue, together with increased disorganization of the fibres may contribute to an increase in AF susceptibility to rupture, impairing NP confinement and allowing herniation [1]. A reduction in Col I in AF tissue has been associated with ageing [1, 11, 16], and with the degeneration level in both goat [44] and human (in vitro study) [17].

Col II stained area in the AF showed a trend to increase in the most herniated tissues, in opposite to Col I, suggesting ECM turnover regarding the collagen composition of the AF. It is possible that this ECM turnover is also accompanied by alterations in the mechanical properties of the tissue and, therefore, it may become more susceptible to herniation, since Col II has a smaller elastic modulus than Col I [45]. Further biomechanical tests should be conducted to evaluate this possibility. Nevertheless, the literature describes a reduction of Col II from non-degenerated to degenerated or herniated IVD, both in AF tissue [11] or whole IVD [18, 19]. In fact, there is a lack of knowledge of the herniation containment level in most of the literature. Additionally, FN stained area was shown to increase from hernias contained by AF to more severe herniation stages. This is in accordance with the literature, in which FN showed to increase from low to high degeneration grades (evaluated by Thompson’s scale) [15], as well as between protrusion and extrusion [18], both in human IVD.

Concurrently, hAF fibrotic markers were also evaluated at the cellular level. In the current work, α-SMA+ cells were absent in all AIS samples, suggesting MF activity in AF tissue only during IVD herniation. α-SMA+ cells have been previously reported to increase from non-degenerated to degenerated IVD, both in human and rat [12]. Hastreiter et al. reported an increase percentage of α-SMA+ cells in extruded tissues, when compared to scoliosis samples [46]. In our study, most of the samples present low levels of α-SMA+ cells (< 20%), in agreement with previous reports (4–15% of α-SMA expression) [46]. However, some heterogeneity between the samples was observed, suggesting that, although a reliable marker for fibrosis in several tissues, α-SMA might not be optimal for IVD herniation.

We have also investigated the expression of MMP12, a pro-fibrotic marker upregulated under inflammatory conditions [12]. MMP12 has been associated with IVD degeneration in a rat model [29] and in human AF [12]. However, although Lv et al. suggested MMP12 as a degeneration marker [12], our study did not show any correlation between MMP12 and the progression of IVD herniation, since MMP12+ cells were observed in most of control samples and absent in some of the herniated samples. Moreover, although MMP12 has been associated with α-SMA, herein our results indicate higher expression of MMP12 than α-SMA [12]. Lastly, we also evaluated the presence of macrophages, important players in tissue fibrosis. We observed macrophage infiltration in a small number of samples and in a low percentage (< 2.0%), distributed within the outer layers of the AF. Moreover, although macrophages were found in some AIS samples, these cells were not present in protused hernias. Previous studies detected CD68+ macrophages in the AF of pig, mice and human [27, 28, 47]. Gronblad et al. highlighted their abundant presence in AF of human herniated tissue, but without analysing differences between hernia types [48]. Our findings are in agreement with previous reports where macrophages were detected in herniated IVD and are believed to be key players in hernia resorption through phagocytosis [3, 49]. Additionally, an evaluation of a possible interaction of age within the comparisons regarding hernia containment was conducted, revealing no influence of this factor in the observed differences. However, no differences were observed between different herniated conditions suggesting that the ECM fibrotic alterations do not correlate directly with the frequency of macrophages present on hAF, indicating that other players might be involved.

This study presents some limitations. The use of thoracic/lumbar discs collected during anterior release surgeries for AIS may not be the ideal control for lumbar discs collected from patients operated for herniated lumbar discs. As in practice IVD samples from healthy donors are almost inaccessible, AIS samples were the most suitable option for control group. However, it has to be considered that although MRI analysis suggests that the NP from scoliosis patients retains in normal conditions of water, the IVD of these conditions might be under abnormal spine load and effects on AF tissue are still not well defined [12, 50]. AIS discs have been described to present sparse elastic fibres with some disorganization in collagen and elastic fibre networks, loss of lamellar structure in the AF and presence of cell clusters [51]. In addition, AIS AF presents a decrease in elastin and Col I and decrease of Col III and Collagen IV [52]. Therefore, caution should be taken when analysing the results since some differences can either be covered or less attenuated [12]. Moreover, a reduced sample size of different hernia types might influence the statistical power of comparisons, but still it was possible to observe significant differences between parameters analysed in the hAF tissues. Although our conclusions have shown to be independent of the age of the patients, we cannot exclude the influence that other external factors (gender, body mass index, smoking habits or other co-morbidities) might have in the analysis. In the future, it would be relevant to address other fibrosis markers for a better understanding of AF failure in IVD herniation.

## Conclusions

To the best of our knowledge, this is the first study that characterizes fibrotic events in the AF tissue of herniated IVD in a systematic way, at distinct stages of hernia containment. This study suggests important alterations in the hAF tissue during herniation that have been associated with tissue fibrosis. Structural changes were observed in the distribution of collagen fibres in herniated AF, in particular their lack of organization in uncontained samples. The stained area of sGAG and Col I were decreased in all the containment levels of herniated AF when compared with AIS AF, while FN and Col II were increased in AF of more advanced stages of IVD herniation. Moreover, α-SMA tends to increase in the AF of uncontained hernias. Hence, our work indicates herniation progression stage as an important parameter to understand the process of tissue fibrosis in hAF and consequently, of AF failure and IVD herniation.

## Supplementary Information

**Additional file 1: Supplementary Materials and Methods. Supplementary Table S1**: Donor information regarding gender, age, Pfirrmann scale and condition. **Supplementary Figure S1**: Image analysis workflow. Schematization of the image analysis process, for both ECM and cellular markers. **Supplementary Figure S2**: Human AF mosaic representation with histological staining Alcian Blue/Picro-Sirius Red. Distinct hernia containment levels are presented, as follows (a) AIS, (b) Protused, (c) Contained and (d) Uncontained. Rectangles indicate the area where staining analysis was further conducted. Scale bar: 500 μm. **Supplementary Figure S3**: Example representation of negative IHC controls for each staining. (a) Collagen I; (b) Collagen II; (c) Fibronectin, scale bar: 100 μm; (d) α-SMA; (e) MMP12, scale bar: 50 μm.

## Data Availability

The data that supports the findings of the present work are available from the corresponding author upon reasonable request.

## Abbreviations

AB: Alcian blue
AF: Annulus fibrosus
AIS: Adolescent idiopathic scoliosis
Col I: Collagen I
Col II: Collagen II
Col III: Collagen III
ECM: Extracellular matrix
FN: Fibronectin
IHC: Immunohistochemistry
IVD: Intervertebral disc
LBP: Low back pain
MFs: Myofibroblasts
MMP: Matrix metalloproteinase
MRI: Magnetic resonance imaging
NP: Nucleus pulposus
PLL: Posterior longitudinal ligament
PSR: Picro-Sirius red
TEM: Transmission electron microscopy
hAF: Human annulus fibrosus
sGAG: Sulphated glycosaminoglycans
α-SMA: Alpha-smooth muscle actin
