# Supplementary material for: Fibrotic alterations in human annulus fibrosus correlate with progression of intervertebral disc herniation
Source: Arthritis Res Ther. 2022 Jan 17;24:25. doi: 10.1186/s13075-021-02690-w (PMC8762926; doi:10.1186/s13075-021-02690-w)
Supplement: Supplementary file 1 — Additional file 1: Supplementary Materials and Methods. Supplementary Table S1: Donor information regarding gender, age, Pfirrmann scale and condition. Supplementary Figure S1: Image analysis workflow. Schematization of the image analysis process, for both ECM and cellular markers. Supplementary Figure S2: Human AF mosaic representation with histological staining Alcian Blue/Picro-Sirius Red. Distinct hernia containment levels are presented, as follows (a) AIS, (b) Protused, (c) Contained and (d) Uncontained. Rectangles indicate the area where staining analysis was further conducted. Scale bar: 500 μm. Supplementary Figure S3: Example representation of negative IHC controls for each staining. (a) Collagen I; (b) Collagen II; (c) Fibronectin, scale bar: 100 μm; (d) α-SMA; (e) MMP12, scale bar: 50 μm. [file 13075_2021_2690_MOESM1_ESM.pdf]

Fibrotic alterations in human annulus fibrosus correlate with progression of intervertebral disc herniation

Supplementary Materials and Methods

Transmission electron microscopy analysis

Fixed hAF tissue was processed for TEM analysis. Samples were washed in PBS and fixed in 2.5% glutaraldehyde (2h) and 2% paraformaldehyde in 0.1 M sodium cacodylate (pH 7.4). After a 30 min wash in 0.1 M sodium cacodylate buffer, the tissue was fixed in 2% (v/v) osmium tetroxide in 0.1 M sodium cacodylate overnight followed by fixation (1% uranyl acetate) overnight. Samples were dehydrated in a gradient series of ethanol solutions: 50% ethanol (10 min), followed by 70%, 80%, 90%, 96%, 100% and propylene oxide (v/v). Further, samples were included in EPON resin in a silicon mould, using gradually increasing series of propylene oxide to EPON as follows: 2:1, 1:1, 1:2 and 0:1 (60 min each). EPON polymerization was performed at 60 °C (48h). Samples were cut (50nm sections) using a diamond knife (Diatome, Hatfield, PA, USA) and recovered to 200 mesh Formvar Ni-grids. Prior to observation, sections were stained with 2 wt% uranyl acetate and saturated lead citrate solution(7 min each). Visualization was performed at 80 kV in a JEOL JEM 1400 microscope (Japan) (5000x and 12000x magnifications). 15-20 images for each sample were analysed (n = 3).

Histological analysis

Dissected AF samples were fixed (10% neutral buffered formalin), embedded in paraffin and sectioned (3 µm). Sections were stained for Alcian Blue (AB) and

Picro-Sirius Red (PSR) to analyse sGAG (blue)/collagens (red) content, respectively. Briefly, samples were dewaxed, rehydrated and incubated in AB solution (pH=1.0, 30 min), washed in tap water, and stained in PSR solution (1 h). Afterwards, a final wash in acidified water (0,5% acetic acid) (30 s) was performed and sections were cleared and mounted with Entellan (Merck).

Immunohistochemistry

Col I, FN, α-SMA, MMP12, Col II and CD68 expression in AF sections was assessed by IHC, using NovolinkTM Polymer Detection Kit (Leica Biosystems, Newcastle, UK), according to manufacturer's instructions. Both optimized antigen retrieval and antibody dilutions are described in Table 1. Briefly, for each staining, slides were dewaxed and rehydrated, followed by antigen retrieval and an endogenous peroxidase and protein block (5 minutes each). Next, sections were incubated with the primary antibody, under the conditions described in Table1. Slides were then incubated in post-primary solution and NovolinkTM Polymer (30 min each). A final incubation with peroxidase-substrate DAB solution (5 minutes) in the dark allowed the revelation of bound antibodies, followed by a hematoxylin staining. Slides were mounted with Entellan.

Supplementary Tables

Table S1: Donor information regarding gender, age, Pfirrmann scale and condition

ID	Gender	Age	Pfirrmann	Disc Level	Condition
**1**	F	45	4	L5-S1	~~Contained by AF~~ Protused
**2**	F	30	4	L5-S1	~~Contained by AF~~ Protused
**3**	F	~~43~~ 44	~~3~~ 4	L5-S1	~~Contained by AF~~ Protused
**4**	M	50	3	L4-L5	~~Contained by AF~~ Protused
**5**	M	~~44~~ 43	~~4~~ 3	L4-L5	~~Contained by AF~~ Protused
**6**	M	41	3	L4-L5	~~Contained by AF~~ Protused
**7**	M	24	4	L4-L5	~~Contained by AF~~ Protused
**8**	M	72	5	L4-L5	~~Contained by AF~~ Protused
**9**	M	56	5	L5-S1	~~Contained by AF~~ Protused
**10**	F	50	3	L4-L5	~~Extruded~~ Uncontained
**11**	F	34	4	L5-S1	~~Extruded~~ Uncontained
**12**	F	50	3	L5-S1	~~Extruded~~ Uncontained
**13**	F	29	4	L5-S1	~~Extruded~~ Uncontained
**14**	F	44	3	L4-L5	~~Extruded~~ Uncontained
**15**	F	37	4	L5-S1	~~Extruded~~ Uncontained
**16**	F	44	4	L5-S1	~~Extruded~~ Uncontained
**17**	M	30	3	L5-S1	~~Extruded~~ Uncontained
**18**	M	56	3	L2-L3	~~Extruded~~ Uncontained
**19**	M	34	3	L4-L5	~~Extruded~~ Uncontained
**20**	M	35	4	L5-S1	~~Extruded~~ Uncontained
**21**	M	35	4	L5-S1	~~Extruded~~ Uncontained
**22**	M	36	4	L5-S1	~~Extruded~~ Uncontained
**23**	F	40	4	L5-S1	~~Contained by PLL~~ Contained
**24**	F	70	3	L4-L5	~~Contained by PLL~~ Contained
**25**	F	47	5	L4-L5	~~Contained by PLL~~ Contained
**26**	F	45	3	L4-L5	~~Contained by PLL~~ Contained
**27**	F	55	3	L5-S1	~~Contained by PLL~~ Contained
**28**	F	71	3	L4-L5	~~Contained by PLL~~ Contained
**29**	F	36	3	L4-L5	~~Contained by PLL~~ Contained
**30**	F	41	4	L4-L5	~~Contained by PLL~~ Contained
**31**	F	39	5	L5-S1	~~Contained by PLL~~ Contained
**32**	F	27	3	L5-S1	~~Contained by PLL~~ Contained
**33**	F	65	4	L3-L4	~~Contained by PLL~~ Contained
**34**	M	38	3	L4-L5	~~Contained by PLL~~ Contained
**35**	M	83	5	L4-L5	~~Contained by PLL~~ Contained
**36**	M	34	3	L5-S1	~~Contained by PLL~~ Contained
**37**	M	47	3	L5-S1	~~Contained by PLL~~ Contained
**38**	M	77	4	L5-S1	~~Contained by PLL~~ Contained
**39**	M	53	4	L4-L5	~~Contained by PLL~~ Contained

**40**	F	16	-	n.a.	~~Scoliosis~~ AIS
**41**	F	21	-	n.a.	~~Scoliosis~~ AIS
**42**	F	16	-	n.a.	~~Scoliosis~~ AIS
**43**	M	15	-	n.a.	~~Scoliosis~~ AIS
**44**	M	15	-	n.a.	~~Scoliosis~~ AIS
**45**	M	19	-	n.a.	~~Scoliosis~~ AIS

F- female; M- male; n.a. – not available ~~AF – Annulus Fibrosus; PLL – Posterior Longitudinal Ligament~~

Supplementary Figures

Figure S1

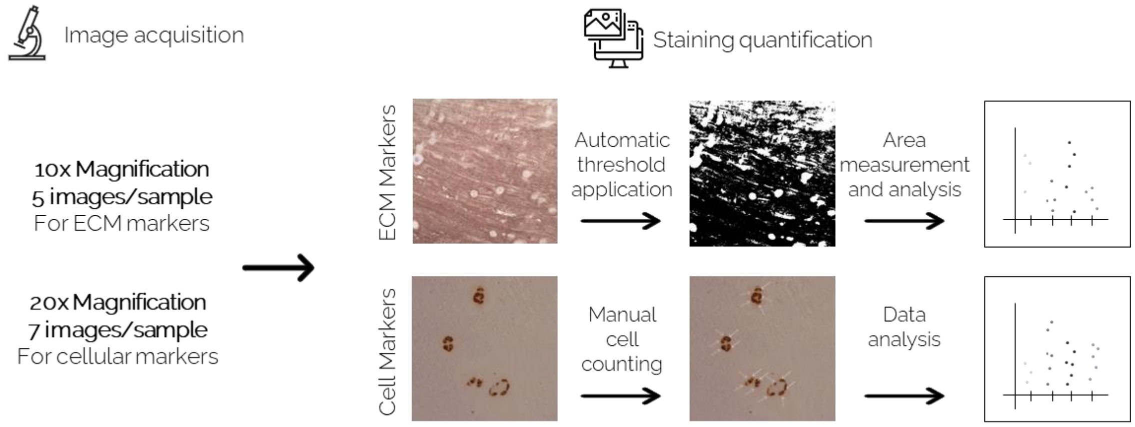

Figure S1: **Image analysis workflow.** Schematization of the image analysis process, for both ECM and cellular markers.

Figure S2

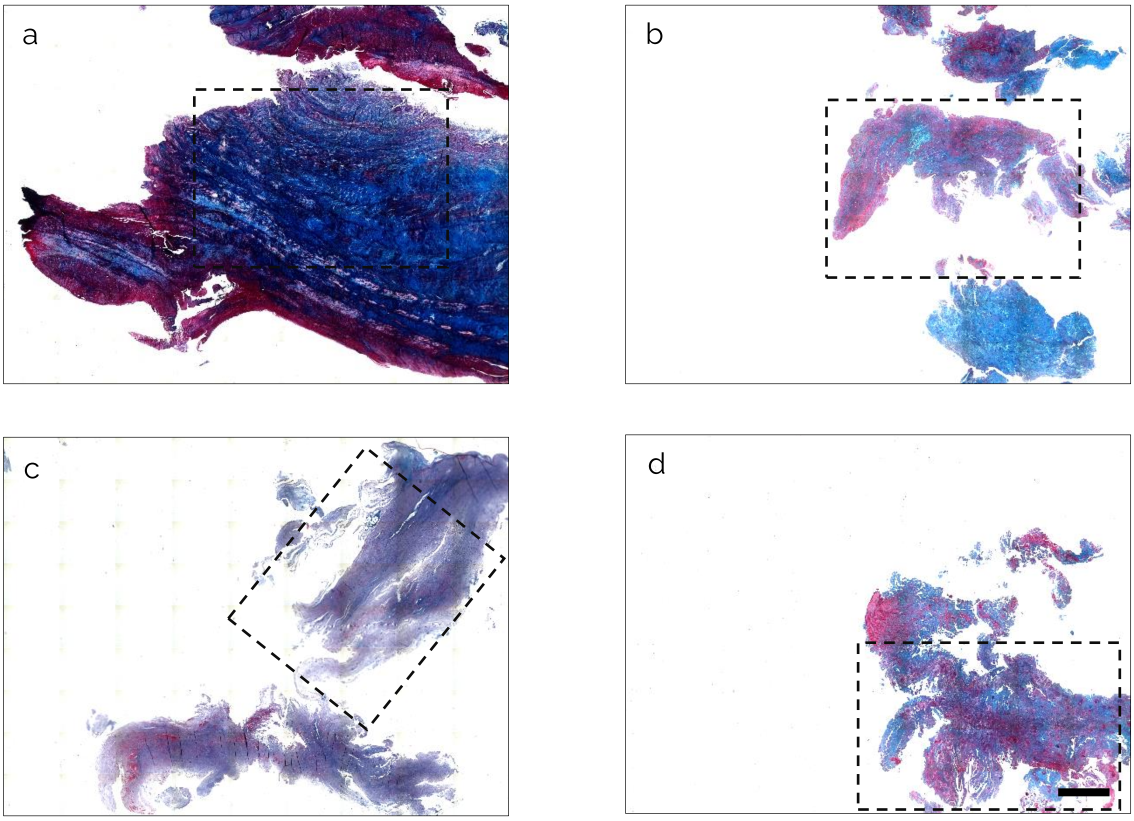

Figure S2: **Human AF mosaic representation with histological staining Alcian Blue / Picro-Sirius Red.** Distinct hernia containment levels are presented, as follows (a) AIS, (b) Protused, (c) Contained and (d) Uncontained. Rectangles indicate the area where staining analysis was further conducted. Scale bar: 500 µm.

Figure S3

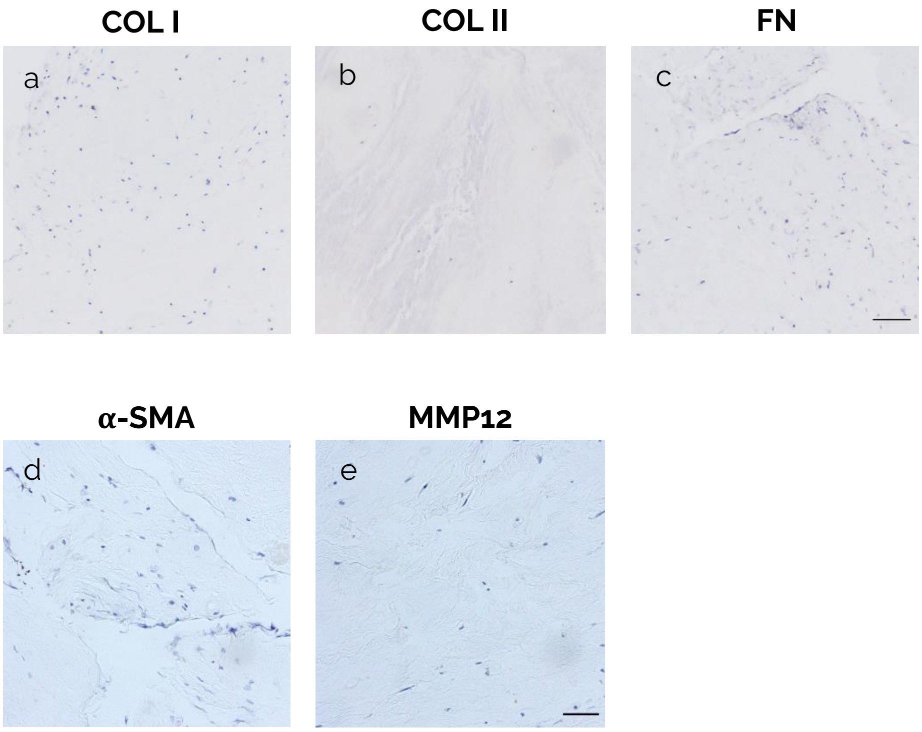

Figure S3: **Example representation of negative IHC controls for each staining.** (a) Collagen I; (b) Collagen II; (c) Fibronectin, scale bar: 100 µm; (d) α-SMA; (e) MMP12, scale bar: 50 µm.
